# Hormonal Interplay of GAs and Abscisic Acid in Rice Germination and Growth Under Low-Temperature Stress

**DOI:** 10.3390/ijms27010181

**Published:** 2025-12-23

**Authors:** Nari Kim, Rahmatullah Jan, Saleem Asif, Sajjad Asaf, Hak Yoon Kim, Kyung-Min Kim

**Affiliations:** 1 Department of Applied Biosciences, Kyungpook National University, Daegu 41566, Republic of Korea; jennynari@hanmail.net; 2 Coastal Agriculture Research Institute, Kyungpook National University, Daegu 41566, Republic of Korea; rehmatbot@yahoo.com (R.J.); saleemasif10@gmail.com (S.A.); 3 Natural and Medical Science Research Center, University of Nizwa, Nizwa 616, Oman; sajadasif2000@gmail.com; 4 Department of Environmental Engineering, Keimyung University, Daegu 42601, Republic of Korea

**Keywords:** abscisic acid (ABA), gibberellic acid (GA_3_), hormonal regulation, rice cultivars, stress tolerance

## Abstract

Seed germination and early growth in rice are critical stages influenced by the hormonal balance between gibberellins (GA) and abscisic acid (ABA), particularly under low-temperature stress. This study investigated the effects of GA_3_ and ABA on seed germination, embryonic growth, gene expression, and biochemical activities in rice cultivars with contrasting tolerance to low temperatures. GA_3_ markedly improved germination in resistant cultivars Nagdong and CNDH77, whereas susceptible cultivars showed minimal improvement, while ABA strongly inhibited germination, especially under higher concentrations. GA_3_ also promoted embryonic growth, with resistant cultivars displaying the longest embryo cells (10.10 µm and 13.49 µm, respectively), whereas ABA suppressed embryonic growth and completely inhibited germination in susceptible cultivars. Upregulation of GA biosynthesis (*OsCPS1* and *OsKS1*) and signaling genes (*OsGID1* and *OsGID2*) in resistant cultivars correlated with enhanced germination and growth, whereas ABA-induced *ABI5* expression suppressed germination, particularly in susceptible cultivars. Hormone quantification confirmed increased endogenous GA_3_ after GA_3_ treatment and reduced ABA levels under ABA treatment. Additionally, GA_3_ modulated ABA signaling genes, upregulating *OSK3*, *ABI3*, *ABI4*, and *ABI5*, while ABA treatment had contrasting effects, particularly between resistant and susceptible cultivars. GA_3_ treatment also enhanced the expression of GA biosynthesis and signaling genes (*OsCPS1*, *OsKS1*, *OsGID1*, and *OsGID2*), whereas ABA treatment upregulated ABA catabolic genes (*OsABA8ox2*). GA_3_ also enhanced amylase activity and sugar-related gene expression, supporting its role in energy mobilization during germination. Conversely, ABA suppressed cell elongation, reducing it to 4.45 µm in CNDH77 under 100 µM ABA. These findings provide valuable insights into the hormonal regulation of rice seed germination and growth under low-temperature stress, offering potential strategies to enhance seed vigor and stress tolerance in rice breeding.

## 1. Introduction

The life cycle of a plant begins with seed germination, which is initiated by the imbibition of water into a dry seed and completed when the radicle emerges from the seed coat. This complex process is regulated by multiple intrinsic and extrinsic factors, including seed dormancy, stored food reserves, temperature, oxygen, light, humidity, and the presence of chemical compounds in the surrounding environment [1]. Seed responses to environmental cues are critical in determining plant species distribution and community structure. Therefore, understanding germination patterns under varying environmental conditions is essential for optimizing seed utilization and improving natural ecosystem management [2,3].

Temperature is a major environmental factor influencing seed germination and early seedling growth in crop plants. In rice, temperatures below 20 °C inhibit germination, slow seedling development, delay tillering, disrupt flowering, and induce panicle sterility, ultimately reducing grain yield [4]. In subtropical regions, unfavorable sowing conditions, such as low temperatures and cold water irrigation, weaken seed vigor and reduce germination rates and speed [5]. These adverse effects contribute to uneven germination, delayed seedling emergence, and, in severe cases, seedling deficiency, compromising early growth and overall plant establishment [6,7]. The impact of low temperatures on rice germination is a major limitation to the adoption of direct seeding techniques, as it significantly affects seedling establishment [5]. Rice germinates optimally between 20 and 33 °C, with 15 °C serving as the threshold for evaluating cold tolerance during germination tests [8]. However, other studies suggest that the ideal temperature range for rice germination is 25–30 °C, and exposure to cold stress is a major abiotic constraint limiting rice productivity in various regions worldwide [9]. Rice is highly sensitive to low temperatures at all growth stages, leading to poor germination, stunted growth, increased plant mortality, and delayed flowering [10]. Cold stress also extends the growth cycle, reduces tillering, and significantly lowers yield [11,12]. In particular, exposure to low temperatures during the booting stage disrupts panicle branching and pollen development, ultimately reducing the number of grains per panicle and the seed-setting rate [12]. Additionally, cold stress alters reproductive structures, impairing anther cell wall development, reducing pollen viability, and increasing sterility, all of which contribute to lower grain production [11,13,14].

Phytohormones play a crucial role in regulating key developmental processes throughout the plant life cycle, including seed maturation, germination, floral transition, and responses to both biotic and abiotic stresses [15]. Among them, abscisic acid (ABA) and gibberellins (GA) are two antagonistic hormones that play opposing but interconnected roles in seed dormancy, germination, and plant development [16,17,18]. The balance between ABA and GA determines whether a seed remains dormant or initiates germination, making their interaction crucial for regulating seed establishment [19]. In highly dormant seeds, ABA accumulates during seed development, reaching peak levels by the time of embryo maturation [20], thereby reinforcing dormancy by inhibiting water uptake and preventing cell-wall loosening two essential processes required for germination [21,22]. At the molecular level, ABA enforces dormancy by inducing Late Embryogenesis Abundant (*LEA*) gene expression and promoting growth arrest by activating the basic leucine zipper transcription factor Abscisic Acid-Insensitive 5 (*ABI5*) [23]. Under unfavorable environmental conditions, ABA levels remain high, suppressing GA biosynthesis and further reinforcing dormancy. However, when conditions become favorable—such as sufficient moisture, light, and optimal temperature—the internal ABA concentration declines, triggering dormancy release [24,25]. ABA catabolism plays a crucial role in this process, as sequential hydroxylation and conjugation reactions progressively degrade ABA, thereby promoting germination [26].

ABA degradation is mediated by the cytochrome P450 family 707 (CYP707A) enzymes, which possess ABA 8′-hydroxylase activity, converting ABA into phaseic acid (PA). PA is subsequently processed into dihydrophaseic acid (DPA) by phaseic reductase and further metabolized into its conjugated forms by glycosyltransferases, completing the ABA degradation pathway [27]. During seed imbibition, ABA levels progressively decline, coinciding with an increase in PA and DPA accumulation, as observed in *Lactuca sativa*, *Arabidopsis thaliana*, and *Hordeum vulgare* seeds [28,29]. This metabolic conversion facilitates the gradual reduction in ABA content, ultimately promoting germination [30].

While ABA reinforces seed dormancy, GA functions as a counteracting force, promoting germination by stimulating cell elongation and mobilizing stored nutrients within the endosperm. The transition from dormancy to germination is tightly regulated by external environmental cues such as light, temperature, and moisture, which activate GA biosynthetic pathways. Under optimal conditions, GA biosynthesis is upregulated, leading to a reduction in ABA levels [31,32,33]. Several transcription factors, including PIL5, BME3, and SPT, regulate GA biosynthesis by enhancing its production in response to germination-promoting conditions [31,32,33]. Cold stratification and light exposure have been shown to stimulate GA biosynthesis, further tipping the hormonal balance in favor of germination. These antagonistic interactions between ABA and GA are central to the regulation of seed dormancy and germination.

The discovery of ABA receptors, particularly the *PYR*/*PYL*/*RCAR* family, has provided significant insights into ABA signaling and its role in abiotic stress responses [34]. In Arabidopsis and rice, these receptors deactivate protein phosphatase 2C (*PP2C*) in the presence of ABA, thereby enabling the activation of SNF1-related protein kinase 2 (*SnRK2*). Activated *SnRK2* then triggers ABRE-binding factors (ABFs), which regulate ABA-responsive genes involved in dormancy maintenance and stress adaptation [35,36]. Mutations in *PYL* genes alter ABA sensitivity, whereas their overexpression enhances stress tolerance, highlighting their importance in seed germination, growth, and adaptation to environmental stresses [37,38]. The interplay between ABA and GA is central to balancing dormancy and germination, with ABA inhibiting water uptake and GA promoting cell expansion and nutrient mobilization. ABA degradation through hydroxylation and conjugation pathways facilitates dormancy release and germination. Furthermore, the discovery of ABA receptors and their signaling mechanisms has deepened our understanding of plant responses to abiotic stress. Therefore, understanding ABA–GA interactions is essential for optimizing seed germination strategies in agriculture, particularly under adverse climatic conditions.

Despite extensive research on ABA and GA interactions in seed dormancy and germination, their precise regulatory mechanisms under low-temperature conditions in rice remain poorly understood. While studies in *Arabidopsis thaliana* suggest that ABA accumulation enforces dormancy and GA counteracts ABA’s inhibitory effects, the extent to which these mechanisms function in rice under cold stress is still unclear. To bridge this knowledge gap, this study investigated the effects of exogenous ABA and GA on rice cultivars with varying cold stress tolerance. The primary objective was to determine how exogenous ABA and GA influence germination under low temperatures and to elucidate their impact on endogenous hormone biosynthesis, amylase activity, and sugar metabolism. This study provides key insights into rice seed responses to cold stress during germination, supporting efforts to enhance cold tolerance in rice and improve the feasibility of direct seeding in low-temperature regions.

## 2. Results

### 2.1. GA_3_ Promotes Germination and Growth, While ABA Inhibits Seed Germination

**Figure 1:**
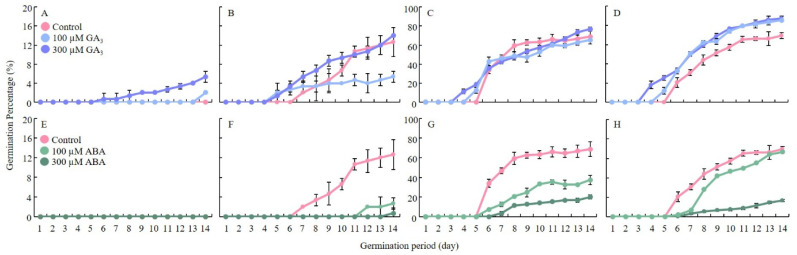
Effects of GA_3_ and ABA on rice seed germination under low-temperature conditions. Seeds were incubated at 15 °C for 14 days. Panels (**A**–**D**) represent GA_3_ treatment, while panels (**E**–**H**) represent ABA treatment. (**A**,**E**) Cheongcheong (susceptible line), (**B**,**F**) CNDH30 (susceptible line), (**C**,**G**) Nagdong (resistant line), and (**D**,**H**) CNDH77 (resistant line). Data are presented as the mean ± standard deviation (SD).

A significant contrast in GP was observed between GA_3_ and ABA treatments, highlighting their opposing roles in seed germination (Figure 1). Seeds treated with 300 µM GA_3_ showed the highest GP, with Nagdong and CNDH77 reaching 76.7% and 87.3%, respectively, by 14 days after imbibition (Figure 1C,D). In contrast, the susceptible varieties Cheongcheong and CNDH30 displayed markedly lower GP, at 5.3% and 14.0%, respectively (Figure 1A,B). Notably, Nagdong and CNDH77 initiated germination on day 4, with a sharp increase in GP between days 5 and 6. Conversely, ABA treatment strongly suppressed germination across all genotypes compared to the control (Figure 1E–H). Under 300 µM ABA, GP was reduced to 20.0% in Nagdong, 16.7% in CNDH77, and a strikingly low 0.7% in CNDH30, while Cheongcheong failed to germinate entirely. Under 100 µM ABA, CNDH77 exhibited the highest GP among all ABA-treated seeds (parental line and CNDH30).

**Figure 2:**
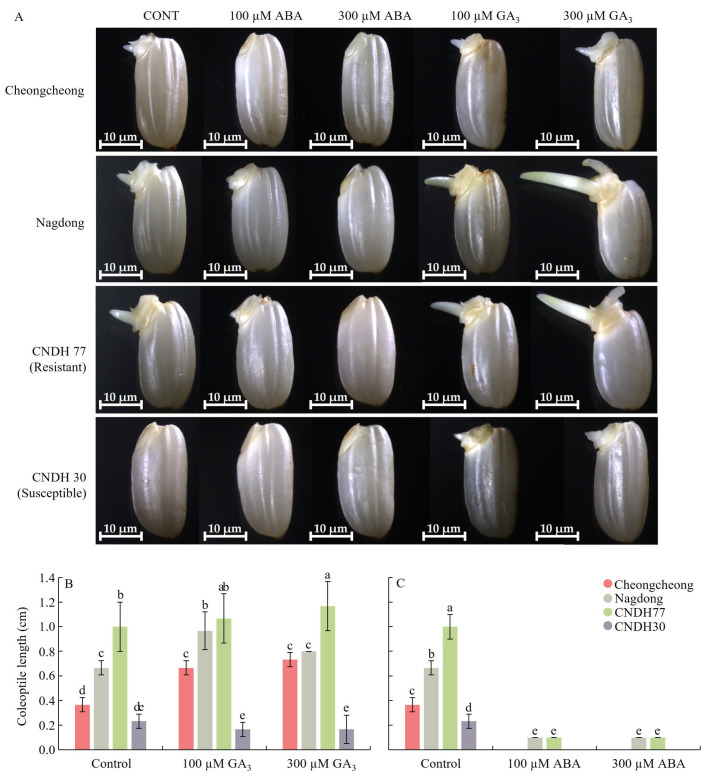
Morphology of rice seed embryos and coleoptile length following GA_3_ and ABA treatment under low-temperature conditions. (**A**) Representative images of rice seed embryos after 4 days of incubation at 15 °C with ABA and GA_3_ treatments. The white line at the bottom of panel (**A**) represents the scale bar (10 μm). (**B**,**C**) Coleoptile length measurements 7 days after imbibition. (**B**) Coleoptile length in GA_3_-treated rice seeds. (**C**) Coleoptile length in ABA-treated rice seeds. Data are presented as the mean ± SD. Different letters above the bars indicate statistically significant differences (*p* < 0.05) based on Duncan’s multiple range test. DMRT was used to compare differences among cultivars at each concentration level. CNDH77 (resistant line), CNDH30 (susceptible line).

GA_3_ treatment also promoted embryonic growth, with Nagdong and CNDH77 producing the longest embryos under 300 µM GA_3_ (Figure S1). Seed germination in 300 µM GA_3_-treated seeds began after 4 days. In contrast, no germination was observed in any genotype treated with 300 µM ABA at the same time point (Figure 1A,E). Similarly, GA_3_ treatment significantly promoted coleoptile elongation. At 100 µM GA_3_, coleoptile length reached 0.7 cm in Cheongcheong, 1.0 cm in Nagdong, and 1.1 cm in CNDH77, while at 300 µM GA_3_, it increased to 0.7 cm, 0.8 cm, and 1.2 cm, respectively (Figure 2B). In contrast, ABA treatment severely restricted coleoptile growth, with Nagdong and CNDH77 reaching only 0.1 cm under both 100 µM and 300 µM ABA, while Cheongcheong and CNDH30 failed to grow even after 7 days after imbibition (Figure 2C). These findings confirm the antagonistic roles of GA_3_ and ABA in seed germination under cold stress, with GA_3_ markedly enhancing germination and early seedling growth, while ABA strongly suppresses these processes. The contrasting responses between resistant and susceptible lines further highlight genotype-specific hormonal sensitivity during cold-induced germination.

### 2.2. GA_3_ and ABA Modulate Endogenous Hormones in Rice Seeds Under Low-Temperature Stress

**Figure 3:**
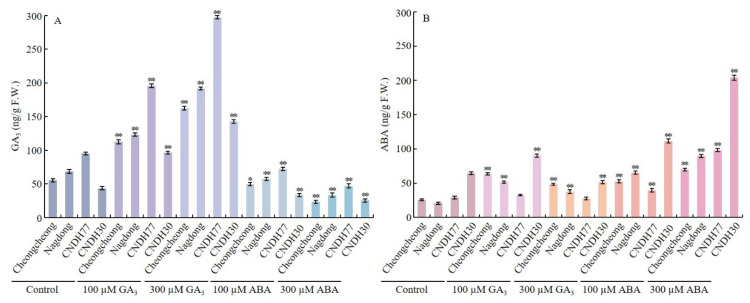
Quantification of endogenous GA_3_ and ABA levels in rice seeds. (**A**) Endogenous GA_3_ levels. (**B**) Endogenous ABA levels. Data are presented as the mean ± SD. Statistical significance was determined by comparing each treatment with its corresponding control within the same cultivar. Asterisks indicate significant differences (* *p* < 0.05 and ** *p* < 0.01) based on the Bonferroni test. CNDH77 (resistant line), CNDH30 (susceptible line).

The interplay between GA_3_ and ABA is crucial, as GA_3_ promotes seed germination and early seedling growth, whereas ABA inhibits these processes while enhancing stress tolerance. To investigate the physiological and biochemical responses of rice seeds to low-temperature stress during germination, we quantified endogenous GA_3_ and ABA levels (Figure 3). GA_3_ treatment significantly increased endogenous GA_3_ levels, with CNDH77 exhibiting 297.35 ng/g under 300 µM GA_3_ and 195.68 ng/g under 100 µM GA_3_, while Nagdong recorded 191.77 ng/g under 300 µM GA_3_, demonstrating the stimulatory effect of GA_3_ treatment on its endogenous accumulation.

In contrast, ABA treatment reduced endogenous ABA levels in Cheongcheong and CNDH30, with concentrations measuring 23.31 ng/g and 25.46 ng/g, respectively, under 300 µM ABA, representing a substantial decline compared to the control (Figure 3A). A clear divergence in endogenous GA_3_ levels was observed between GA_3_- and ABA-treated seeds, with GA consistently leading to higher endogenous GA_3_ accumulation than ABA-treated seeds.

Regarding endogenous ABA, the control condition measured 64.44 ng/g, whereas CNDH30 exhibited a striking increase under 300 µM and 100 µM ABA treatments, reaching 204.02 ng/g and 111.43 ng/g, respectively. Notably, ABA levels increased in both GA_3_ and ABA-treated seeds across all varieties compared to the control, except in CNDH77 and CNDH30 under the 300 µM GA_3_ treatment, where ABA did not show a substantial increase (Figure 3B). In summary, GA_3_ treatment increased endogenous GA_3_ levels, while ABA treatment elevated endogenous ABA, highlighting their antagonistic roles during cold-induced germination. The distinct hormonal profiles among genotypes highlight the importance of GA_3_ and ABA balance in regulating germination and stress responses under low-temperature conditions.

### 2.3. GA_3_ and ABA Modulate Key ABA Signaling Genes During Rice Seed Germination

**Figure 4:**
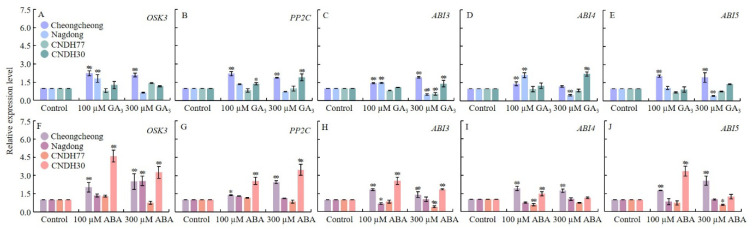
Relative expression of seed germination and ABA signaling-related genes in response to low-temperature (15 °C) treatment. Panels (**A**–**E**) represent GA_3_-treated conditions, while panels (**F**–**J**) represent ABA-treated conditions. Data are presented as the mean ± SD. Statistical significance was determined by comparing each treatment with its corresponding control within the same cultivar. Asterisks indicate significant differences (* *p* < 0.05 and ** *p* < 0.01) based on the Bonferroni test. CNDH77 (resistant line), CNDH30 (susceptible line).

To investigate the regulatory role of GA_3_ and ABA in ABA signaling during rice seed germination, we assessed the relative expression of key ABA-responsive genes: serine/threonine protein kinase (*OSK3*), protein phosphatase type 2C (*PP2C*), abscisic acid-insensitive genes (*ABI3*, *ABI4*, and *ABI5*) (Figure 4).

GA_3_ treatment significantly upregulated *OSK3* in Cheongcheong at both 100 µM and 300 µM concentrations, while Nagdong showed upregulation only at 100 µM GA_3_ (Figure 4A). Similarly, *PP2C* expression increased significantly in Cheongcheong and CNDH30 under both GA_3_ treatments (Figure 4B). For *ABI3* and *ABI4*, 100 µM GA_3_ induced expression in Cheongcheong and Nagdong, while 300 µM GA_3_ significantly upregulated *ABI3* in Cheongcheong and CNDH30 and *ABI4* in CNDH30 (Figure 4C,D). Notably, *ABI5* expression was significantly increased only in Cheongcheong under both GA_3_ treatments (Figure 4E).

ABA treatment strongly influenced gene expression patterns. *OSK3* was significantly upregulated in Cheongcheong and CNDH30 under both 100 µM and 300 µM ABA, whereas Nagdong exhibited a significant upregulation only at 300 µM ABA (Figure 4F). *PP2C* expression increased significantly in Cheongcheong and CNDH30 under both ABA concentrations (Figure 4G). Furthermore, *ABI3* and *ABI4* showed consistent upregulation in Cheongcheong and CNDH30 for both ABA treatments, whereas CNDH77 exhibited downregulation (Figure 4H,I). For *ABI5*, Cheongcheong and CNDH30 displayed significant upregulation under 100 µM ABA, with Cheongcheong also showing increased expression at 300 µM ABA, while CNDH77 exhibited downregulation under 300 µM ABA treatment (Figure 4J). These results demonstrated that GA_3_ and ABA treatment differentially modulated the expression of key ABA responsive genes, with susceptible lines showing strong upregulation of *OSK3*, *PP2C*, *ABI3*, *ABI4*, and *ABI5* under both hormones. In contrast, resistant lines, particularly CNDH77, exhibited weaker or even repressed expression under ABA treatment. These contrasting transcriptional responses highlight genotype-dependdent ABA signaling dynamic and reinforce the antagonistic regulatory roles of GA_3_ and ABA during cold-induced seed germination.

### 2.4. GA_3_ and ABA Regulate ABA Metabolism in Rice Seeds Under Low-Temperature Germination

**Figure 5:**
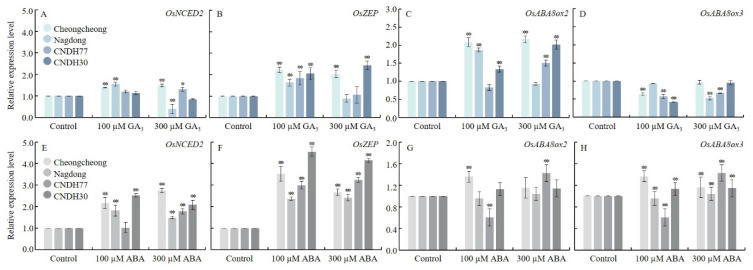
Relative expression of ABA metabolism-related genes during seed germination at 15 °C. Panels (**A**–**D**) represent GA_3_-treated conditions, while panels (**E**–**H**) represent ABA-treated conditions. Data are presented as the mean ± SD. Statistical significance was determined by comparing each treatment with its corresponding control within the same cultivar. Asterisks indicate significant differences (* *p* < 0.05 and ** *p* < 0.01) based on the Bonferroni test. CNDH77 (resistant line), CNDH30 (susceptible line).

To examine how GA_3_ and ABA influence ABA metabolism, we analyzed the expression levels of key genes involved in ABA biosynthesis—*Oryza sativa* 9-cis-epoxycarotenoid dioxygenase 2 (*OsNCED2*) and *O. sativa* zeaxanthin epoxidase (*OsZEP*)—and ABA catabolism—*O. sativa* abscisic acid 8′-hydroxylase 2 (*OsABA8ox2*) and *O. sativa* abscisic acid 8′-hydroxylase 3 (*OsABA8ox3*), which are involved in ABA catabolism (Figure 5).

Under GA_3_ treatment, expression of *OsNCED2* was significantly upregulated in Cheongcheong and Nagdong under 100 µM GA_3_, and in Cheongcheong and CNDH77 under 300 µM GA_3_ (Figure 5A). *OsZEP* expression was significantly increased across all genotypes except Nagdong and CNDH77 under 300 µM GA_3_ (Figure 5B). Furthermore, *OsABA8ox2* expression was significantly enhanced in Cheongcheong, Nagdong, and CNDH30 under both 100 µM and 300 µM GA_3_ (Figure 5C). However, *OsABA8ox3* expression was downregulated in all genotypes under both GA_3_ concentrations (Figure 5D).

Under ABA treatment, *OsNCED2* expression was significantly upregulated in all genotypes under both 100 µM and 300 µM ABA, except for CNDH77 under 100 µM ABA (Figure 5E). Similarly, *OsZEP* expression was significantly upregulated in all genotypes under both ABA concentrations (Figure 5F). Regarding ABA catabolism, *OsABA8ox2* and *OsABA8ox3* exhibited similar expression patterns in response to ABA treatments. Specifically, Cheongcheong and CNDH30 exhibited higher expression levels than Nagdong and CNDH77 at 100 µM ABA, whereas CNDH77 showed significant upregulation of both genes under 300 µM ABA (Figure 5G,H). These results show that GA_3_ and ABA treatments distinctly modulated ABA metabolic pathways. GA_3_ generally promoted ABA biosynthesis gene expression while variable affecting catabolic genes, whereas ABA strongly induced both biosynthesis and catabolism genes across the genotypes. These contrasting patterns highlight genotype-specific regulation of ABA metabolism during cold-induced germination.

### 2.5. GA_3_ and ABA Alter GA Transduction Genes During Rice Seed Germination Under Low Temperatures

**Figure 6:**
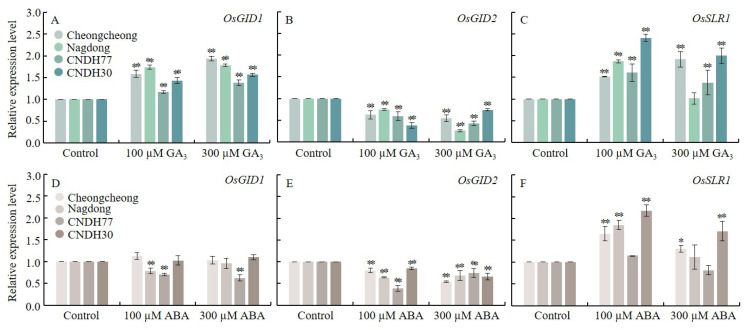
Relative expression of GA signaling-related genes during seed germination at 15 °C. Panels (**A**–**C**) represent GA_3_-treated conditions, while panels (**D**–**F**) represent ABA-treated conditions. Data are presented as the mean ± SD. Statistical significance was determined by comparing each treatment with its corresponding control within the same cultivar. Asterisks indicate significant differences (* *p* < 0.05 and ** *p* < 0.01) based on the Bonferroni test. CNDH77 (resistant line), CNDH30 (susceptible line).

To investigate the effects of GA_3_ and ABA on GA transduction-related genes in rice seedlings, we analyzed the relative expression levels of key GA signaling genes, including *OsGID1* and *OsGID2* (GA receptors) and *OsSLR1*, which encodes the DELLA protein, a GA signal transduction inhibitor (Figure 6).

Under GA_3_ treatment, *OsGID1* was significantly upregulated in all lines at both 100 µM and 300 µM concentrations. In contrast, *OsGID2* exhibited notable downregulation at both concentrations (Figure 6A,B). Meanwhile, *OsSLR1* was significantly upregulated in all lines except Nagdong under both 100 µM and 300 µM GA_3_ (Figure 6C).

In contrast, ABA treatment led to a significant downregulation of *OsGID1* and *OsGID2* at both 100 µM and 300 µM concentrations (Figure 6D,E). Notably, *OsSLR1* was significantly upregulated under 100 µM ABA in Cheongcheong, Nagdong, and CNDH30, whereas 300 µM ABA treatment further increased *OsSLR1* expression in Cheongcheong and CNDH30 (Figure 6F). These results show that GA_3_ treatment promoted GA signaling by inducing *OsGID1*, and modulating *OsSLR1*, whereas ABA suppresses GA perception and enhanced DELLA expression. These opposing effects highlights the contrasting regulation of GA transduction under cold stress.

### 2.6. GA_3_ and ABA Regulate GA Biosynthesis Genes in Rice Seeds

**Figure 7:**
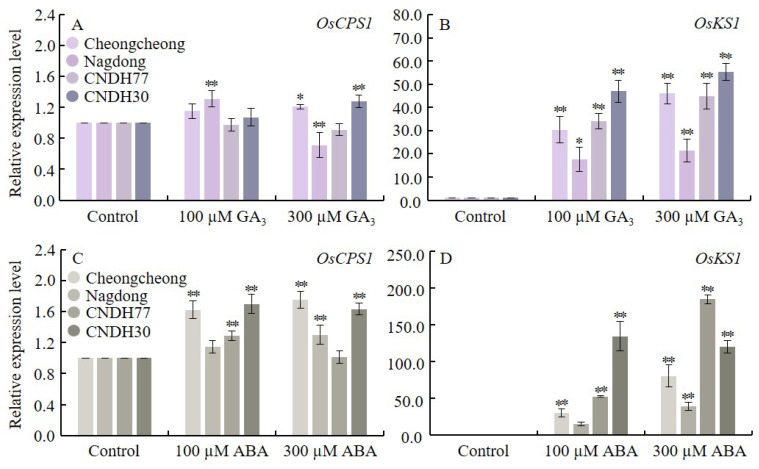
Relative expression of GA biosynthesis-related genes during seed germination at 15 °C. Panels (**A**,**B**) represent GA_3_-treated conditions, while panels (**C**,**D**) represent ABA-treated conditions. Data are presented as the mean ± SD. Statistical significance was determined by comparing each treatment with its corresponding control within the same cultivar. Asterisks indicate significant differences (* *p* < 0.05 and ** *p* < 0.01) based on the Bonferroni test. CNDH77 (resistant line), CNDH30 (susceptible line).

To assess the impact of GA_3_ and ABA on GA biosynthesis in rice seedlings, we examined the relative expression levels of *O. sativa* ent-copalyl diphosphate synthase 1 (*OsCPS1*) and *O. sativa* ent-kaurene synthase 1 (*OsKS1*), which are involved in the initial steps of GA biosynthesis (Figure 7).

The results showed that *OsCPS1* expression was significantly upregulated under 100 µM GA_3_ in Nagdong, and under 300 µM GA_3_ in Cheongcheong and CNDH30. In contrast, Nagdong exhibited a significant reduction in *OsCPS1* expression under 300 µM GA_3_ compared with the control (Figure 7A). The relative expression of OsKS1 increased significantly in all lines under both GA_3_ concentrations (Figure 7B).

Under ABA treatment, *OsCPS1* expression was elevated in all lines, with Cheongcheong and CNDH30 showing the highest induction at both ABA concentrations (Figure 7C). Likewise, *OsKS1* expression was higher under both ABA concentrations relative to the control, with the strongest induction observed in CNDH77 under 300 µM ABA, followed by CNDH30 under 100 µM ABA (Figure 7D).

Overall, these findings indicate that both GA_3_ and ABA positively regulate the expression of key diterpenoid biosynthesis genes (*OsCPS1* and *OsKS1*) during germination, with line-specific responses reflecting genotype-dependent hormonal sensitivity.

### 2.7. GA_3_ and ABA Modulate Sugar Transport and Accumulation in Rice Seed During Germination Under Low Temperatures

**Figure 8:**
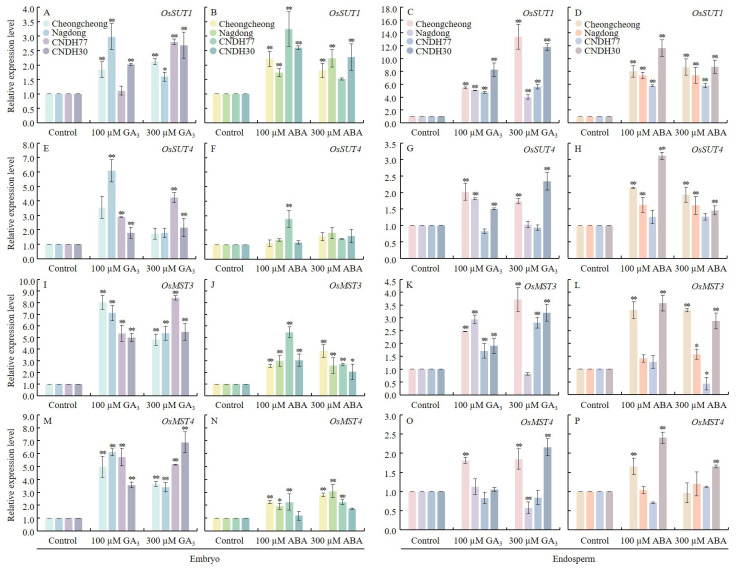
Expression of sugar metabolism-related genes in rice seeds following GA_3_ and ABA treatment at 15 °C. Panel (**A**–**D**) represents expression of *OsSUT1* in embryo and endosperm under GA_3_ and ABA treatment respectively. Panel (**E**–**H**) represents expression of *OsSUT4* in embryo and endosperm under GA_3_ and ABA treatment respectively. Panel (**I**–**L**) represents expression of *OsMST3* in embryo and endosperm under GA_3_ and ABA treatment respectively. Panel (**M**–**P**) represents expression of *OsMST4* in embryo and endosperm under GA_3_ and ABA treatment respectively. Data are presented as the mean ± SD. Statistical significance was determined by comparing each treatment with its corresponding control within the same cultivar. Asterisks indicate significant differences (* *p* < 0.05 and ** *p* < 0.01) based on the Bonferroni test. CNDH77 (resistant line), CNDH30 (susceptible line).

To examine the effects of GA_3_ and ABA on sugar biosynthesis and transport in rice, we assessed the expression of sucrose transporter genes (*OsSUT1* and *OsSUT4*) and monosaccharide transporter genes (*OsMST3* and *OsMST4*) in both the seed embryo and endosperm (Figure 8).

In the embryo, *OsSUT1* was upregulated in all varieties under both GA_3_ and ABA treatments, with the most pronounced increases observed in Nagdong (100 µM GA_3_) and CNDH77 (100 µM ABA) (Figure 8A,B). *OsSUT4* was significantly upregulated in embryo of all lines under GA_3_ treatments, particularly in Nagdong (100 µM GA_3_) and CNDH77 (300 µM GA_3_), while only CNDH77 showed upregulation under ABA treatment (Figure 8E,F). Similarly, *OsMST3* in embryo, were strongly upregulated in GA_3_-treated seeds, while in ABA treated seeds, CNDH77 showed the highest *OsMST3* expression under 100 µM ABA (Figure 8I,J). Similarly, *OsMST4* in embryo showed enhanced upregulation in all the concentrations of both the hormones (Figure 8 M,N).

In the endosperm, *OsSUT1* was significantly upregulated across all lines under both GA_3_ and ABA treatments, with Cheongcheong and CNDH30 exhibiting the highest expression levels compared to control (Figure 8C,D). A similar trend was observed for *OsSUT4* expression, with the exception of CNDH77, which showed reduced expression under both GA_3_ concentrations (Figure 8G,H). For *OsMST3* expression was highest in Cheongcheong and CNDH30 under 300 µM GA_3_, while both the concentrations of ABA treatment showed highest expression level in Cheongcheong and CNDH30 Compared to control (Figure 8K,L). Similarly, *OsMST4* expression in the endosperm increased in Cheongcheong under 100 µM GA_3_, while under 300 µM GA_3_, both Cheongcheong and CNDH30 showed significant upregulation compared with the control (Figure 8O). In ABA-treated seeds, Cheongcheong and CNDH30 exhibited significantly higher expression under 100 µM ABA, and CNDH30 also showed elevated expression under 300 µM ABA relative to the control (Figure 8P). Regarding sugar content, glucose levels significantly increased in Cheongcheong and Nagdong under both GA_3_ treatments, whereas glucose content decreased in all lines under both ABA treatments (Figure S2A,B). Lactose content increased significantly in Nagdong under both GA_3_ concentrations and also under both ABA treatments compared to the control (Figure S2C,D). Mannitol levels increased in CNDH30 under both GA_3_ and ABA treatments, but decreased in other lines under both conditions (Figure S2E,F). Overall results show that GA_3_ broadly enhanced sugar transporter gene expression in both the embryo and endosperm, while ABA showed more genotype-specific induction. GA_3_ increased glucose accumulation, whereas ABA reduced it across genotypes. These results indicate that GA_3_ promotes sugar mobilization to support germination, whereas ABA generally suppresses sugar availability under cold stress.

### 2.8. GA_3_ and ABA Regulate Amylase Activity and Related Gene Expression During Rice Seed Germination

**Figure 9:**
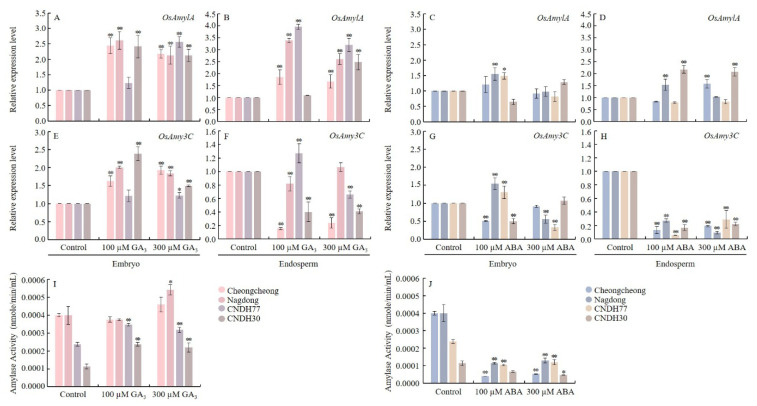
Expression of amylase activity-related genes and variations in amylase activity in rice seeds following GA_3_ and ABA treatment at 15 °C. Panel (**A**–**D**) represents expression of *OsAmy1A* in embryo and endosperm under GA_3_ and ABA treatment respectively. Panel (**E**–**H**) represents expression of *OsAmy3C* in embryo and endosperm under GA_3_ and ABA treatment respectively. Panel (**I**,**J**) represents Amylase activity under GA_3_ and ABA treatment respectively. Data are presented as the mean ± SD. Statistical significance was determined by comparing each treatment with its corresponding control within the same cultivar. Asterisks indicate significant differences (* *p* < 0.05 and ** *p* < 0.01) based on the Bonferroni test. CNDH77 (resistant line), CNDH30 (susceptible line).

To examine the regulatory effects of GA_3_ and ABA on amylase activity, we analyzed the expression of α-amylase genes, *O. sativa* α-amylase 1A (*OsAmy1A*) and *O. sativa* α-amylase 3C (*OsAmy3C*), along with changes in amylase activity (Figure 9).

In the embryo and endosperm, *OsAmy1A* and *OsAmy3C* were upregulated under both 100 µM and 300 µM GA_3_ treatments across all rice lines (except CNDH30in endosperm under 100 µM GA_3_), compared to the control (Figure 9A,B). Under ABA treatment, *OsAmy1A* in embryo was significantly upregulated only in Nagdong and CNDH77 at 100 µM ABA (Figure 9C), while in endosperm it showed significant upregulation in Nagdong and CNDH30 at 100 µM ABA and Cheongcheong and CNDH30 at 300 µM ABA relative to control (Figure 9C,D).

The relative expression of *OsAmy3C* in the embryo was upregulated in all lines under both GA_3_ concentrations (Figure 9E). In contrast, its expression in the endosperm was generally downregulated, with the exception of CNDH77 under 100 µM GA_3_ and Nagdong under 300 µM GA_3_, which exhibited slight induction compared to the control plants (Figure 9F). However, under both ABA concentrations, *OsAmy3C* expression in the embryo and endosperm was generally downregulated (Figure 9G,H). Notably, Nagdong and CNDH77 exhibited significant upregulation in the embryo under 100 µM ABA compared with the control plants (Figure 9G). Amylase activity analysis revealed that CNDH77 and CNDH30 exhibited significant increases under 100 µM GA_3_ treatment, whereas Nagdong, CNDH77, and CNDH30 showed significant increases under 300 µM GA_3_ compared with the control plants (Figure 9I). In contrast, amylase activity was significantly reduced in all lines under both 100 µM and 300 µM ABA treatments compared to the control (Figure 9J). These results showed that GA_3_ strongly promoted α-amylase gene expression and activity, enhancing starch breakdown, whereas ABA variably affected transcript level but consistently suppressed amylase activity, highlighting its inhibitory role during germination.

### 2.9. GA_3_ and ABA Modulate Cell Elongation in Rice Seed During Germination Under Low Temperatures

**Figure 10:**
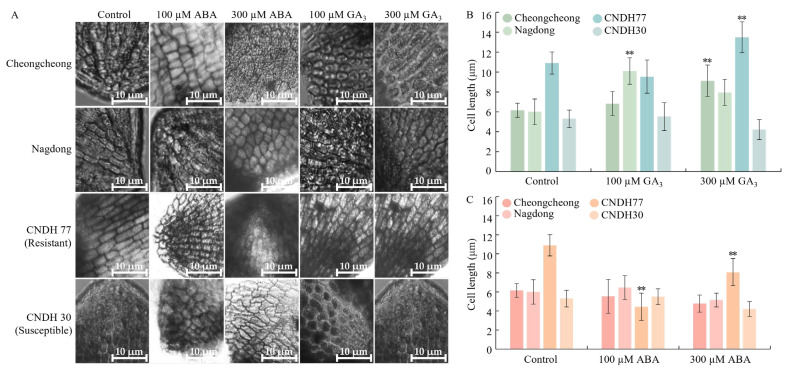
Microscopic analysis and cell length measurement of rice embryo cells under GA_3_ and ABA treatment at 15 °C. (**A**) Representative images of rice embryo cells subjected to control, 100 µM ABA, 300 µM ABA, 100 µM GA_3_, and 300 µM GA_3_ treatments. White line at right bottom of panel (**A**) represent scale bars = 10 μm. (**B**,**C**) Cell length measurements in rice embryo cells 5 days after coleoptile emergence under GA_3_ and ABA treatment respectively. For each treatment and genotype, cell length was measured from 10 randomly selected cells, including small, medium, and large cells, using ImageJ software (v1.8.0) to capture natural cell size variation. Data are presented as the mean ± SD. Statistical significance was determined by comparing each treatment with its corresponding control within the same cultivar. Asterisks indicate significant differences (** *p* < 0.01) based on the Bonferroni test. CNDH77 (resistant line), CNDH30 (susceptible line).

To investigate the roles of GA_3_ and ABA in regulating cell elongation during rice seed germination under low-temperature conditions, we measured cell length in rice seed embryos following hormone treatments (Figure 10).

Treatment with both GA_3_ concentrations generally promoted embryo cell elongation compared with the control plants. However, CNDH77 under 100 µM GA_3_ and CNDH30 under 300 µM GA_3_ did not show this increase. These trends are clearly illustrated in the representative micrographs and quantified measurements shown in Figure 10A,B. In contrast, ABA treatment at both concentrations inhibited embryo cell elongation in all lines, with the exception of Nagdong under 100 µM ABA, which showed enhanced elongation relative to the control. These patterns are evident in the representative micrographs and corresponding quantitative measurements presented in Figure 10A,C. Overall, these results demonstrate that GA_3_ promotes embryo cell elongation during rice seed germination under low-temperature conditions, whereas ABA predominantly suppresses this process. The contrasting responses among specific lines (CNDH77, CNDH30, and Nagdong) highlight genotype-dependent hormonal sensitivity, suggesting that variations in GA_3_ and ABA responsiveness contribute to differential germination performance under low-temperature stress.

## 3. Discussion

Rice is a staple food for a significant portion of the global population. However, its cultivation faces challenges from environmental constraints, particularly low-temperature stress during seed germination. Low temperatures hinder germination, reduce seedling vigor, and ultimately limit crop establishment. This sensitivity discourages direct seeding in cooler regions, leading to increased labor costs and resource inputs. Therefore, understanding the hormonal regulation of germination under low-temperature stress is crucial for improving rice cultivation in these environments. Importantly, low temperature shifts the hormonal balance toward ABA dominance by enhancing ABA biosynthesis and signaling, creating a molecular state that actively suppress GA-mediated germination pathways.

Our study investigated the germination patterns of low-temperature-resistant and susceptible rice cultivars under exogenous ABA and GA_3_ applications. The results indicate that GA_3_ treatment significantly enhances germination and coleoptile elongation under cold conditions, particularly in resistant cultivars. Conversely, susceptible cultivars exhibited a weaker response to GA_3_, suggesting reduced GA_3_ sensitivity and a stronger dormancy mechanism under cold stress. Recent studies have reported that a 10 µM GA_3_ treatment increased the GP at 15 °C to 38.6%, representing a 238.6% improvement compared to untreated seeds [39]. Additionally, ABA levels were reported to be higher in seeds exposed to 15 °C than in those at 30 °C, while GA_3_ application reduced ABA content under low-temperature conditions. Our findings align with these observations, demonstrating that exogenous GA_3_ application increases endogenous GA_3_ levels in resistant lines. This suggests that resistant cultivars enhance GA biosynthesis or signaling more efficiently than susceptible ones, improving germination under low-temperature stress. This may reflect ability of resistant lines to rapidly activate GA receptors and downstream transcriptional components, thereby overcoming the ABA-enforced dormancy induced by cold.

Furthermore, GA_3_-treated seeds exhibited increased coleoptile cell size compared to ABA-treated seeds, suggesting the direct role of GA_3_ in enhancing cell expansion. Conversely, ABA application increased endogenous ABA levels in susceptible cultivars, reinforcing dormancy mechanisms and exacerbating cold sensitivity. These results support the idea that resistant and susceptible cultivars exhibit differential hormonal regulation—resistant lines favor GA_3_-mediated germination, while susceptible lines are more responsive to ABA-induced dormancy under low temperatures. The earlier germination observed in resistant cultivars likely reflects enhanced GA signaling and metabolic readiness to overcome dormancy under cold stress. However, hormonal levels cannot fully explain the antagonistic regulation between ABA and GA, Thus, we additionally analyzed the transcriptional activity of ABA-responsive genes (*ABI3*, *ABI4*, *ABI5*, *PP2C*, *OSK3*) and GA-responsive genes (*OsGID1*, *OsGID2*, *OsSLR1*), providing a more mechanistic demonstration of the hormonal interplay during cold-induced germination inhibition. Our results reveal that low temperature strengthens ABA signaling by evaluating ABA-responsive transcription factors and *PP2C* regulations, which collectively suppress GA-mediated growth pathways and enforce dormancy.

ABA treatment significantly suppressed germination and coleoptile growth across all cultivars, with the susceptible cultivar being the most affected. This suggests that susceptibility to low temperatures is linked to heightened ABA sensitivity, leading to a stronger inhibition of germination-related processes. Previous studies have shown that chilling stress elevates ABA levels while inhibiting GA_3_ accumulation, thereby suppressing germination. However, exogenous GA_3_ application has been reported to mitigate the inhibitory effects of low temperatures on germination [40]. Our findings further support this, as GA_3_ treatment enhanced germination and coleoptile elongation, particularly in resistant cultivars, while ABA reinforced dormancy mechanisms. The contrasting responses between resistant and susceptible cultivars underscore the critical role of the ABA–GA_3_ balance in regulating cold tolerance during germination. Resistant cultivars demonstrated greater responsiveness to GA_3_, suggesting a more efficient GA biosynthesis or signaling mechanism that facilitates germination despite low-temperature stress. In contrast, susceptible cultivars exhibited stronger ABA-mediated inhibition, further reinforcing dormancy under cold conditions. This differential hormonal regulation highlights the potential for breeding or biotechnological approaches to enhance cold tolerance by modulating ABA–GA_3_ interactions. Understanding these hormonal dynamics is essential for developing rice varieties with improved low-temperature germination, particularly in direct seeding systems where rapid and uniform seedling establishment is crucial for successful crop production. By selecting or engineering cultivars with enhanced GA_3_ responsiveness and reduced ABA-mediated dormancy, rice production can be optimized for cooler climates, reducing the need for labor-intensive seedling transplantation and ensuring stable yields under variable environmental conditions. At the mechanistic level, our data indicate that the resistant cultivars are able to attenuate ABA signaling more effectively, allowing GA-mediated transcriptional programs to dominate even under cold stress.

To further investigate the hormonal regulation of seed germination, we examined the expression patterns of key ABA (*OSK3*, *PP2C*, *ABI3*, *ABI4*, and *ABI5*) and GA (*OsGID1*, *OsGID2*, and *OsSLR1*) signaling pathway genes under low-temperature conditions. These genes play essential roles in stress regulation, particularly during seed dormancy and germination. In a previous study, QTL analysis identified *OSK3*, *PP2C*, and *PYL* as key genes regulating seed germination under low temperatures [41]. In the present study, we found that GA_3_ treatment significantly upregulated *OSK3*, *PP2C*, *ABI3*, and *ABI4* in susceptible varieties, while *ABI5* was enhanced only in Cheongcheong. This transcriptional evidence supports the concept that hormonal interplay is mediated through changes in genes expression, rather than hormone content alone. Notably, GA3-induced downregulation of the DELLA repressor *OsSLR1* in resistant lines provides further mechanistic support for the enhanced GA sensitivity observed at the physiological level. Della repression under GA application represents a key control point, as Della proteins act as central repressors of GA signaling therefore their suppression in resistant lines release GA-responsive genes required for cell elongation and reserve mobilization. Conversely, ABA treatment strongly induced *OSK3*, *PP2C*, *ABI3*, and ABI4 in susceptible varieties but led to ABI3 and ABI4 downregulation in the resistant cultivar CNDH77. Interestingly, *ABI5* exhibited a dose-dependent response, increasing in Cheongcheong and CNDH30 at 100 µM ABA but downregulated in CNDH77 at 300 µM ABA. Overall, the expression levels of these genes were higher under ABA treatment compared to GA_3_ treatment, indicating hypersensitivity to ABA during seed germination under low-temperature conditions. These findings align with studies in Arabidopsis, where *ABI1*, *ABI2*, and *PP2C* genes exhibit hypersensitivity during germination [42]. Several studies have demonstrated that *ABI3*, *ABI4*, and *ABI5* encode transcription factors that regulate ABA-responsive genes, playing a crucial role in germination and dormancy regulation [23,43,44]. The ABA signaling pathway consists of *PYL* receptors, *PP2C* negative regulators, and *SnRK2* positive regulators [45]. ABA binding to *PYLs* suppresses *PP2C* activity, triggering *SnRK2* activation and the induction of ABA-responsive genes [46,47]. Among these regulators, *ABI3* and *ABI4* modulate *ABI5* expression, fine-tuning germination responses under stress conditions [48,49]. Taken together, these genes expression patterns suggest the low temperature promotes a transcriptional cascade that amplifies ABA responses and suppresses GA signaling, with resistant cultivars partially escaping this suppression through targeted modulation of ABI and APP2C components.

Furthermore, exogenous GA_3_ and ABA applications differentially regulated GA signaling-related genes during rice seed germination under low-temperature conditions. GA_3_ treatment significantly upregulated *OsGID1*, while ABA suppressed its expression. Similarly, *OsGID2* expression was reduced under ABA treatment, whereas *OsSLR1* expression was enhanced under both GA_3_ and ABA treatments at low temperatures. However, in the 300 µM GA_3_-treated resistant line (CNDH77), *OsSLR1* expression was significantly lower than that in the susceptible line. This is consistent with previous findings that, in GA signal transduction, GID1 binds to GA to facilitate the degradation of the DELLA protein SLR1, thereby promoting GA responses. Under low-temperature conditions, *OsSLR1* is upregulated, suppressing GA signaling [50]. Our findings align with a previous study reporting that at 15 °C, *OsGID1* and *OsGID2* expression decreased but was restored upon GA_3_ application [39]. Additionally, GA_3_ and ABA application under low temperatures differentially regulated ABA biosynthesis (*OsNCED2* and *OsZEP*) and catabolism (O*sABA8ox2* and *OsABA8ox3*) genes during seed germination. Our results showed that low temperatures induced the expression of *OsNCED2* and *OsZEP* under both GA_3_ and ABA treatments, but expression levels were significantly higher in ABA-treated seeds. In contrast, *OsABA8ox2* expression was higher in GA_3_-treated plants, while *OsABA8ox3* expression was suppressed under low-temperature conditions. In ABA-treated seeds, *OsABA8ox2* and *OsABA8ox3* exhibited irregular expression patterns, likely due to excessive ABA accumulation. The *ABA8ox* genes play a crucial role in ABA degradation, which is essential for seed germination, as low expression of ABA catabolic genes (*OsABA8ox1*, *OsABA8ox2*, and *OsABA8ox3*) strongly inhibits germination [51,52]. It is presumed that the irregular expression of *OsABA8ox2* and *OsABA8ox3* in ABA-treated seeds results from excessive ABA accumulation, disrupting normal degradation pathways. These patterns highlights a mechanistic model where low temperature stabilize ABA accumulation and signaling, while GA_3_ partially restores germination by reactivating ABA catabolism and suppressing DELLA accumulation.

ABA homeostasis is essential for germination and is maintained through a dynamic balance between biosynthesis and degradation. In the ABA biosynthesis pathway, *ZEP* and *NCED* are key regulatory genes, with *ZEP* converting zeaxanthin into violaxanthin, and *NCED* catalyzing the conversion of violaxanthin and neoxanthin into xanthoxin, a rate-limiting step in ABA production [53,54,55]. *NCED* gene expression is directly linked to ABA accumulation. For instance, Arabidopsis seeds overexpressing *AtNCED6* exhibited higher ABA levels during imbibition, effectively preventing germination [56]. Similarly, our study demonstrated that ABA-treated seeds exhibited significantly higher *OsNCED2* and *OsZEP* expression compared to GA_3_-treated seeds, indicating that low-temperature stress induces greater ABA accumulation when seeds are exposed to ABA rather than GA_3_. These findings provide deeper insights into the hormonal regulation of seed germination under low temperatures, emphasizing the critical role of ABA–GA_3_ balance in determining cold tolerance. Understanding these regulatory mechanisms will aid in developing rice cultivars with enhanced cold tolerance, improving seedling establishment, and promoting direct seeding in cooler environments. Thus, the combined patterns of ABA biosynthesis, catabolism, and signaling genes provide a mechanistic framework showing how low temperature enforces ABA dominance and restricts GA action at multiple regulatory layers.

Based on combined physiological, transcriptional, and metabolic analysis, we generally propose a mechanistic model explaining how low temperature enforce ABA and GA regulation during rice seed germination. Under low temperature, ABA treatemtn strongly upregulated ABA biosynthesis genes *OsNCED2* and *OsZEP*, while concurrently suppression or destabilizing ABA catabolic genes *OsABA8ox2* and *OsABA8ox3*, leading to sustained ABA accumulation. Such cold-induced enhancement of Aba biosynthesis and suppression of ABA degradation is a conserved mechanism underlying stress-induced dormancy, as described for ABA-GA antagonism during seed germination [19,57]. This elevated ABA pool activated core ABA signaling components, including *OSK3* and *PP2C*, and induced ABA-responsive transcription factors *ABI3*, *ABI4*, and *ABI5*, particularly in low temperature susceptible lines. The upregulation of these ABA signaling genes reflects a cold-induced reinforcement of dormancy programs, as *ABI3*, *ABI4*, and *ABI5* are known as central regulators that suppress germination related genes expression and maintain embryonic quiescence through ABA-dependent transcriptional cascades [19]. In contrast, GA_3_ treatment partially counteracted this ABA-dominant state by enhancing the expression of ABA catabolic genes especially *OsABA8ox2*, thereby promoting ABA degradation and restoring hormonal homeostasis, consistent with previous models in which GA antagonizes ABA accumulation to permit germination [19].

At the GA regulatory level, low temperature suppressed GA biosynthesis and signaling by limiting GA responsiveness rather than hormone availability alone. ABA treatment downregulated the GA receptor *OsGID1* and reduced *OsGID2* expression, while simultaneously maintain the expression of the DELLA repressor *OsSLR1*, resulted in sustained repression of GA-mediated growth responses. The accumulation of OsSLR1 under ABA and cold conditions represents a key molecular bottleneck, as DELLA protein act as central repressor of GA signaling and inhibit GA-responsive transcription and cell elongation in multiple plant species [57]. Conversely, GA_3_ treatment significantly upregulated *OsGID1* and promoted GA perception, while reducing *OsSLR1* expression in resistant lines, indicating enhanced DELLA degradation. This GA-dependent removal of DELLA repression is a hallmark mechanism through which GA promotes germination and early seedling growth under both optimal and stressed conditions [19,57]. This GA-induced repression of DELLA proteins mechanistically explains the increased GA sensitivity and improve germination observed in resistant lines under low temperature. Collectively, these transcriptional patterns demonstrate that low temperature activates a multilayered ABA-centered regulatory cascade, enhancing ABA biosynthesis and signaling while suppressing GA perception and response which enforcing dormancy. GA_3_ application alleviates this repression by restoring ABA catabolism, reactivating GA receptor signaling, and releasing DELLA-mediated growth inhibition. This antagonistic ABA-GA regulatory framework closely aligns with previously proposed conceptual models of hormone crosstalk during seed germination and abiotic stress responses [19,57]. Resistant lines exhibited greater plasticity in this hormonal network, allowing them to attenuate Aba signaling and sustain GA-driven transcriptional and metabolic programs necessary for successful germination under cold stress.

Seed germination relies heavily on starch degradation as a primary energy source. During this process, starch in the endosperm is broken down into soluble sugars, which are then transported to the embryo to support growth and energy production. Our study examined sugar regulation in germinating rice seeds under low-temperature conditions following GA_3_ and ABA application. Our findings revealed that GA_3_-treated seeds exhibited increased glucose and lactose levels, while mannitol levels were reduced compared to control plants. In contrast, ABA-treated seeds exhibited a reduction in glucose and mannitol levels under low-temperature conditions. A previous study showed that GA_3_ application enhances sugar accumulation in seeds under cold stress, whereas low temperatures reduce amylase activity, thereby limiting starch hydrolysis into soluble sugars [39]. Similarly, Hussain et al. (2016) reported that cold stress significantly decreases soluble sugar content in rice seeds [58]. Efficient seed germination necessitates sugar transport into the embryo; however, low temperatures hinder this process, restricting soluble sugar availability [59]. Our investigation into sugar transporter gene expression revealed that *OsSUT1*, *OsSUT4*, *OsMST3*, and *OsMST4* were upregulated in both the embryo and endosperm under GA_3_ and ABA treatments during low-temperature germination. Notably, *OsSUT1* was more highly expressed in the endosperm, while *OsSUT4*, *OsMST3*, and *OsMST4* were predominantly expressed in the embryo, particularly in GA_3_-treated seeds. These results align with previous research showing that sucrose transporter genes (*OsSUT1*, *OsSUT2*, and *OsSUT4*) and monosaccharide transporter genes (*OsMST3*, *OsMST4*, and *OsMST6*) exhibit higher expression in seeds germinating at 15 °C than 30 °C, with GA_3_ treatment further enhancing their expression under cold stress [39]. However, [59] reported that exposure to 20 °C inhibited sugar transport by suppressing *OsSUT4* expression. This discrepancy may be attributed to differences in germination stages or temperature variations [60]. The enhancement of sugar transporter expression under GA_3_ indicates that GA signaling directly promotes carbon allocation to the embryo, while ABA restricts metabolic flux, reinforcing its inhibitory effects on germination.

During seed germination, high sugar content reduces osmotic pressure and prevents cell death under abiotic stress conditions [61]. Our study demonstrated that GA_3_ application enhances soluble sugar content and amylase activity under low-temperature conditions, whereas ABA application reduces amylase activity and sugar accumulation. Further investigation confirmed that GA_3_ application significantly upregulated *OsAmy1A* and *OsAmy3C* expression in the embryo, while only *OsAmy1A* was induced in the endosperm. Conversely, ABA-treated seeds exhibited a slight increase in *OsAmy1A* expression in both tissues but showed a significant reduction in *OsAmy3C* expression. Notably, a slight induction of *OsAmy3C* was observed in the embryo of resistant lines (Nagdong and CNDH77) under 100 µM ABA treatment. The observed reduction in amylase activity under ABA treatment, despite increased *OsAmy* gene expression, presents a paradox. One possible explanation is that ABA suppresses amylase activity by activating phospholipase D (PLD), which produces phosphatidic acid (PPA), an amylase inhibitor [62]. This suggests that while ABA may upregulate certain amylase genes, post-transcriptional or enzymatic regulatory mechanisms ultimately inhibit its activity, reinforcing dormancy under low-temperature conditions. In contrast, our findings indicate that GA_3_ application enhances amylase activity, promoting starch hydrolysis into soluble sugars and facilitating seed germination under cold stress. Amylase is a central enzyme in starch hydrolysis within the endosperm, and its expression is highly responsive to GA levels. This highlights its pivotal role in regulating seed metabolism and mobilizing energy for germination [63,64]. Together, these results show that GA_3_ reactivates the metabolic machinery required for reserve mobilization, whereas ABA maintains metabolic repression through both transcriptional and post-transcriptional inhibition of amylase activity.

Mechanistically, this study contributes several important new insights into the hormonal and molecular regulation of rice germination under low-temperature stress. Our finding demonstrated that resistant low-temperature resistant cultivars exhibit markedly higher GA sensitivity than susceptible cultivars, sported by stronger induction of the GA receptor and more effective repression of the DELLA protein *OsSLR1*. In contrast, low-temperature susceptible lines show enhanced ABA accumulation and stronger activation of ABA-core signaling genes, including *ABI3*, *ABI4*, *ABI5*, and *PP2C*, which collectively reinforce ABA-mediated seed dormancy. Importantly, the results reveal that low temperature suppress ABA catabolism through the downregulation of ABA8ox genes, leading to excessive ABA accumulation, whereas GA_3_ application help restore ABA homeostasis. Furthermore, we show that GA_3_ enhances amylase activity and sugar transporter expression, promoting metabolic readiness for germination, while ABA exerts a multilayered suppression of carbohydrate mobilization. Together, these findings support a comprehensive mechanism model in which low temperature reinforces ABA dominance and suppresses GA signaling at both hormonal and transcriptional levels, whereas GA_3_ alleviate these inhibitory layers to promote successful germination.

## 4. Materials and Methods

### 4.1. Plant Materials and Seed Germination Treatments

This study utilized four rice cultivars: Cheongcheong, Nagdong, CNDH30, and CNDH77. Cheongcheong (Indica subspecies) and Nagdong (Japonica subspecies) served as the parental lines, while CNDH30 and CNDH77 represent Cheongcheong-Nagdong double haploid (CNDH) lines developed in our laboratory, the Plant Molecular Breeding Lab at Kyungpook National University South Korea. Screening of the CNDH population identified CNDH77 as resistant and CNDH30 as susceptible, whereas the parental lines exhibited contrasting responses, with Cheongcheong being susceptible and Nagdong resistant.

For seed sterilization, seeds were treated with 70% ethanol for 1 min, followed by three washes with double-distilled water (DDW). Next, they were sterilized with 3% sodium hypochlorite for 10 min with continuous shaking on a shaker, then rinsed three times with DDW to remove residual disinfectants [65].

Sterilized seeds (30 per group) were placed on double-layered filter paper in Petri plates and subjected to exogenous phytohormone treatments. GA_3_ and ABA were applied at concentrations of 100 µM and 300 µM, each, with 4 mL of solution per plate. These concentrations were selected on the basis of pre-screening. Control seeds received only DDW. To maintain hormone exposure, an additional 1 mL of the respective hormone solution was applied to each treatment group every 2 days.

Seed germination was monitored daily through visual inspection, and the GP was recorded accordingly. The experiment was conducted under low-temperature conditions (15 °C).

### 4.2. Seed Germination Percentage Measurement

A seed was considered germinated when the radicle visibly emerged and reached a length of at least 2 mm. The number of germinated seeds was recorded daily for 14 days across all treatment groups. GP (%) was calculated using the formula reported previously by [66]:

$$\mathrm{GP}\%\;=\;\left(\frac{Total\;number\;of\;germinated\;seeds}{Total\;number\;of\;seeds\;tested}\right)\times \;100$$

For each rice line, 30 seeds per replicate were used, with three biological replicates performed to ensure statistical robustness.

### 4.3. Quantification of GA_3_ and ABA

To assess GA_3_ and ABA levels in rice seeds during germination under low-temperature conditions, 200 mg seed samples were collected one week after GA_3_ and ABA treatments. Samples were collected in triplicate to ensure statistical accuracy.

For ABA quantification, the Abscisic Acid ELISA Kit (LifeSpan BioSciences, Newark, CA, USA) was used, following the manufacturer’s protocol. Similarly, for GA_3_ analysis, the Gibberellic Acid ELISA Kit (LifeSpan BioSciences, Newark, CA, USA) was used, following the manufacturer’s protocol.

### 4.4. Sugar Content, Coleoptile Length, and Cell Size Measurement

To determine the sugar content, germinated rice seeds from each treatment group were collected and analyzed following the protocol outlined by [67], with minor modifications. Briefly, 100 mg of freeze-dried rice seeds were ground into a fine powder using liquid nitrogen and homogenized in 500 µL of distilled water. The homogenate was then filtered through a 0.45 µm syringe filter to remove insoluble debris. Soluble sugars, including glucose, lactose, and mannitol, were quantified using high-performance liquid chromatography (HPLC) (Model Prominence, Shimadzu Co., Tokyo, Japan) equipped with a Sugar-Pak I column (Ø 6.5 × 300 mm, Waters Co., Milford, MA, USA). HPLC conditions included a mobile phase of 0.01 M Ca-EDTA (50 mg/L in distilled water), a flow rate of 0.5 mL/min, a column temperature of 90 °C, an injection volume of 20 µL, and detection via a refractive index (RI) detector.

Coleoptile length was measured 7 days after germination at 15 °C. Additionally, coleoptile tip cell size was assessed at the same time point. Microscopic images of the coleoptile cells were captured, and cell size was quantitatively analyzed using ImageJ software for precise measurement.

### 4.5. Amylase Activity Assay

Amylase activity was measured using the Amylase Activity Assay Kit (MAK009, Sigma-Aldrich, Burlington, MA, USA), following the manufacturer’s protocol. Powdered tissue samples (100 mg) were homogenized in 0.5 mL of Amylase Assay Buffer and then centrifuged at 13,000 rpm for 10 min at room temperature. The supernatant was collected for enzymatic analysis.

For the assay, 1–50 µL of each sample was added to the wells of a 96-well microplate, and the total volume was adjusted to 50 µL with Amylase Assay Buffer. The reaction was initiated by adding 100 µL of the Master Reaction Mix, prepared by combining equal volumes (50 µL) of Amylase Assay Buffer and Amylase Substrate Mix, per reaction well. The assay included sample, standard, and positive control wells.

Absorbance at 405 nm (A_405_) was recorded after an initial incubation of 2–3 min (T_initial_), followed by continuous measurements at 5 min intervals at 25 °C. Data collection continued until the absorbance of the most active sample approached or exceeded the highest standard (20 nmol/well). The final absorbance reading [(A_405_)_final_] was determined as the penultimate measurement, ensuring it remained within the linear range of the standard curve. The corresponding time point (T_final_) was recorded for enzymatic activity calculations.

### 4.6. RNA Isolation and Quantitative Real-Time PCR (qRT-PCR) Analysis

Total RNA was extracted from rice seeds 5 days after germination using the TRIzol method [68]. RNA was isolated separately from the embryo and endosperm to facilitate gene expression analysis in both tissues. The extracted RNA was quantified, and its concentration was adjusted to 100 ng/µL using RNase-free water (Qiagen, Venlo, The Netherlands).

cDNA synthesis was performed using the UltraScript 2.0 cDNA Synthesis Kit (PCR Biosystems, Wayne, PA, USA), following the manufacturer’s instructions. qRT-PCR was conducted using the StepOnePlus Real-Time PCR System (Fisher Scientific, Hampton, NH, USA) to assess gene expression levels. Each reaction was prepared in a final volume of 20 µL, comprising 10 µL of 2X Real-Time PCR Master Mix, 1 µL each of forward and reverse primers (20 pmol/µL), 1 µL of cDNA, and nuclease-free water to adjust the final volume.

The GA3 and ABA-related genes analyzed in this study such as *OsNCED2*, *OsZEP*, *OsABA8ox2*, *OsGID1*, *OsGID2*, *OsSLR1*, *OsCPS1*, *OsKS1*, *OsSUT1*, *OsSUT4*, *MST3*, *MST4*, *AmyA*, and *Amy3c*, were selected based on previous reports identifying their central role in hormone biosynthesis, signaling, and carbohydrate mobilization during rice seed germination under low-temperature stress [39]. In particular, genes selection was guided by the comprehensive study by [39], which demonstrated the involvement of these genes in GA, ABA regulation, and sugar transport processes during cold affected germination.

The qRT-PCR cycling conditions were as follows: an initial denaturation at 95 °C for 2 min, followed by 40 cycles of denaturation at 95 °C for 20 s, annealing and extension at 60 °C for 40 s, and a final extension at 72 °C for 5 min. *OsActin* was used as the internal control, and all reactions were performed in triplicate. Relative gene expression levels were determined using the 2^−∆∆CT^ method. The primer sequences used in this study are listed in Table S1. To verify amplification specificity, melt curve analyses were performed for all primer pairs using embryo and endosperm cDNA samples. Each gene exhibited a single sharp peak with no evidence of primer-dimers or non-specific products, confirming the specificity and reliability of the qRT-PCR reactions. Representative melt curves are provided in Figure S3.

### 4.7. Statistical Analysis

Data analysis was performed using SPSS software (IBM SPSS Statistics, version 22, Armonk, NY, USA) and GraphPad Prism software (version 5.01, GraphPad Software, San Diego, CA, USA). All experiments included three independent biological replicates to ensure statistical reliability. Results are presented as mean ± standard deviation (SD) to reflect data variability. Statistical significance was determined using Duncan’s multiple range test and Bonferroni post hoc tests, and differences between groups were considered significant at *p* < 0.05.

## 5. Conclusions

This study provides fascinating evidence of the intricate hormonal interplay between GA and ABA in regulating rice seed germination under low-temperature stress. Our findings reveal that GA plays a crucial role in enhancing seed germination, embryonic growth, and cell elongation, particularly in stress tolerant cultivars, while ABA strongly suppresses these processes, especially in susceptible cultivars. The differential expression of GA and ABA related genes further highlights their dynamic influence on stress adaptation. The significant upregulation of GA biosynthesis and signaling genes, coupled with increased amylase activity, highlights GA’s role in mobilizing and promoting vigorous seedling establishment. In contrast, ABA not only reduced endogenous GA levels but also triggered the expression of catabolic and sugar transport genes, leading to impaired and delayed germination. These findings emphasize the fine-tuned hormonal balance for optimal seed performance under adverse environmental conditions. By unraveling the molecular and biochemical mechanisms underlying GA and AB-mediated germination responses, this study offers valuable insights for rice breeding programs. Harnessing these hormonal pathways could cover the way for developing high vigor rice varieties with enhanced resilience to low-temperature stress, ultimately contributing to sustainable rice production in challenging climates.

## Data Availability

The original contributions presented in this study are included in the article/Supplementary Materials. Further inquiries can be directed to the corresponding author(s).

## Supplementary Materials

The following supporting information can be downloaded at: https://www.mdpi.com/article/10.3390/ijms27010181/s1.
