# Supplementary material for: Hormonal Interplay of GAs and Abscisic Acid in Rice Germination and Growth Under Low-Temperature Stress
Source: Int J Mol Sci. 2025 Dec 23;27(1):181. doi: 10.3390/ijms27010181 (PMC12785965; doi:10.3390/ijms27010181)
Supplement: Supplementary file 1 [file ijms-27-00181-s001.zip › ijms-4033512-supplementary.pdf]

Supplementary Materials

Table S1. List of primers used for qRT-PCR experiments.

Primer name	Forward<br>Reverse	Sequence (5’ to 3')
OsActin	F	TGAATCTGGTCCAGGCATCG
	R	TGGGACGCATGCAAACAATC
OSK3	F	GCATGGTTTCTGTGACACACC
	R	TGACGCAAGATCCAAGCTGT
PP2C	F	GACAGTGGCGAGACTGAACA
	R	TTAGACACAACGAGGCCACC
ABI3	F	GTGGAGGTGATCGAGAAGGA
	R	GGCTGCTGTTCCAGTAGGAG
ABI4	F	GTTGCAGCTGCTGTGTCTTC
	R	CTTGAGGAAGAGATCGAACCA

ABI5	F	CAAGGCGGTCCTATGATGTT
	R	ATCCAGGACTCACGACAACC
OsAmy1A	F	TTTCGGTCCTCATCGTCCTCC
	R	TCCACGACTCCCAGTTGAATC
OsAmy3C	F	AAGCATTCCACCACAATGAGC
	R	AGGAAGTTGTACCACCCACC
OsSUT1	F	CCACCTCGGTAGAAGAGAATAA
	R	CCATTCATTACACACTAATTACCAA
OsSUT4	F	TTTGGCTGAGCAGAACACCA
	R	ATGTCATTCGGGCAGAGCTT
OsMST3	F	CTTGTGTTCATCCCCGTGTA
	R	TTGGCCAAACAAGAAACACA
OsMST4	F	CATCATCTCCTGCATCATGG
	R	CAGGCCCTGGTTGTCATACT

OsCPS1	F	TCAAGAGACACCGCCAGTTC
	R	ACAGTGCATGACCCTGGATG
OsKS1	F	GAAGCTGAATGGCAGAGGAC
	R	CTGTTCAGCTTTCCCTCCAG
OsGID1	F	CAAGAGCCTCATCATCGTGT
	R	CATTGGACAACCTTGACGTG
OsGID2	F	CTATCTCAGAGCTGGACACT
	R	CCTCCTGTTTCCGACAAATC
OsSLR1	F	CGTGAAGAGATCGACGACTG
	R	ATGAAATCCAGTTCGGTTCG
OsNCED2	F	GGTATGGAAACGAGGATAGTGGTT
	R	TGCTTATTGTTGTGCGAGAAGTTC
OsZEP	F	ACCTGGCAAGCACTGAAAGT
	R	TCCTCGTGAACCTTTCCATC

OsABA8ox2	F	CTACTGCTGATGGTGGCTGA
	R	CCCATGGCCTTTGCTTTAT
OsABA8ox3	F	AGTACAGCCCATTCCCTGTG
	R	ACGCCTAATCAAACCATTGC

Supplementary Figures

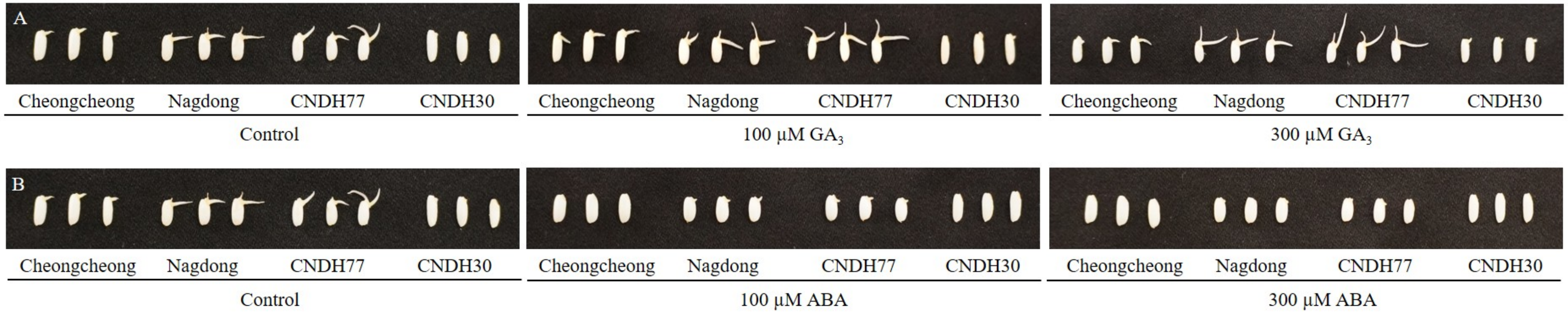

Figure S1. Phenotypes of rice seed germination subjected to control, 100 μM GA_3, 300 μM GA_3, 100 μM ABA, and 300 μM ABA at 15°C ten days after imbibition. (A, B) GA_3 and ABA treatment, respectively. CNDH 77(resistant line), CNDH30 (susceptible line).

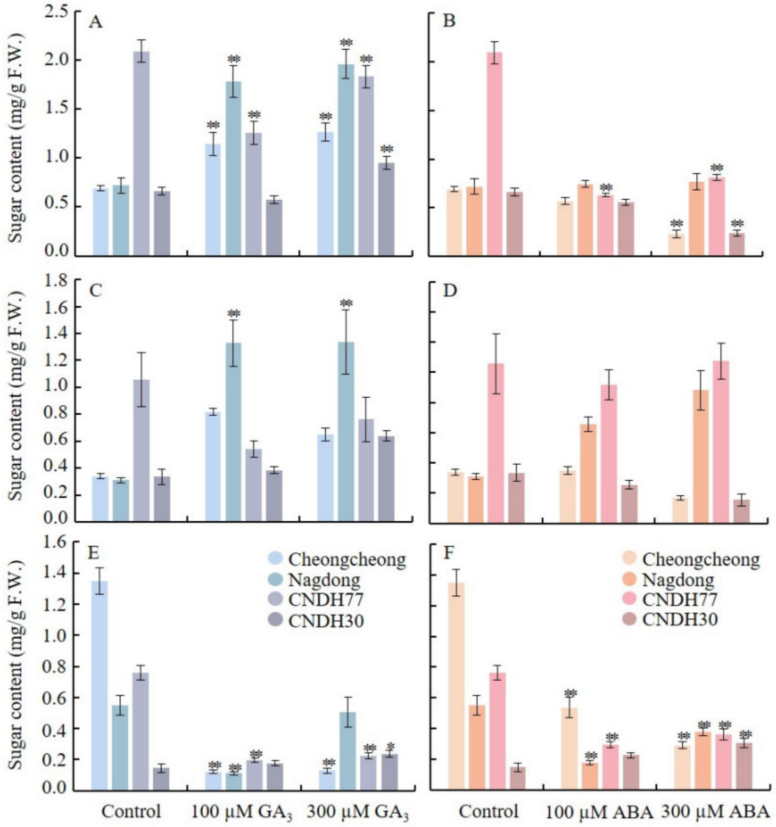

Figure S2. Sugar contents in rice seeds after exogenous GA_3 and ABA treatment at 15°C. (A) Glucose contents in GA_3-treated rice seeds. (B) Glucose contents in ABA-treated rice seeds. (C) Lactose contents in GA_3-treated rice seeds. (D) Lactose contents in ABA-treated rice seeds. (E) Mannitol contents in GA_3-treated rice seeds. (F) Mannitol contents in ABA-treated rice seeds. Data are shown as the mean ± standard deviation, and asterisks show a significant difference (* $p < 0.05$, ** $p < 0.01$) analyzed by the Bonferroni test. CNDH 77(resistant line), CNDH30 (susceptible line).

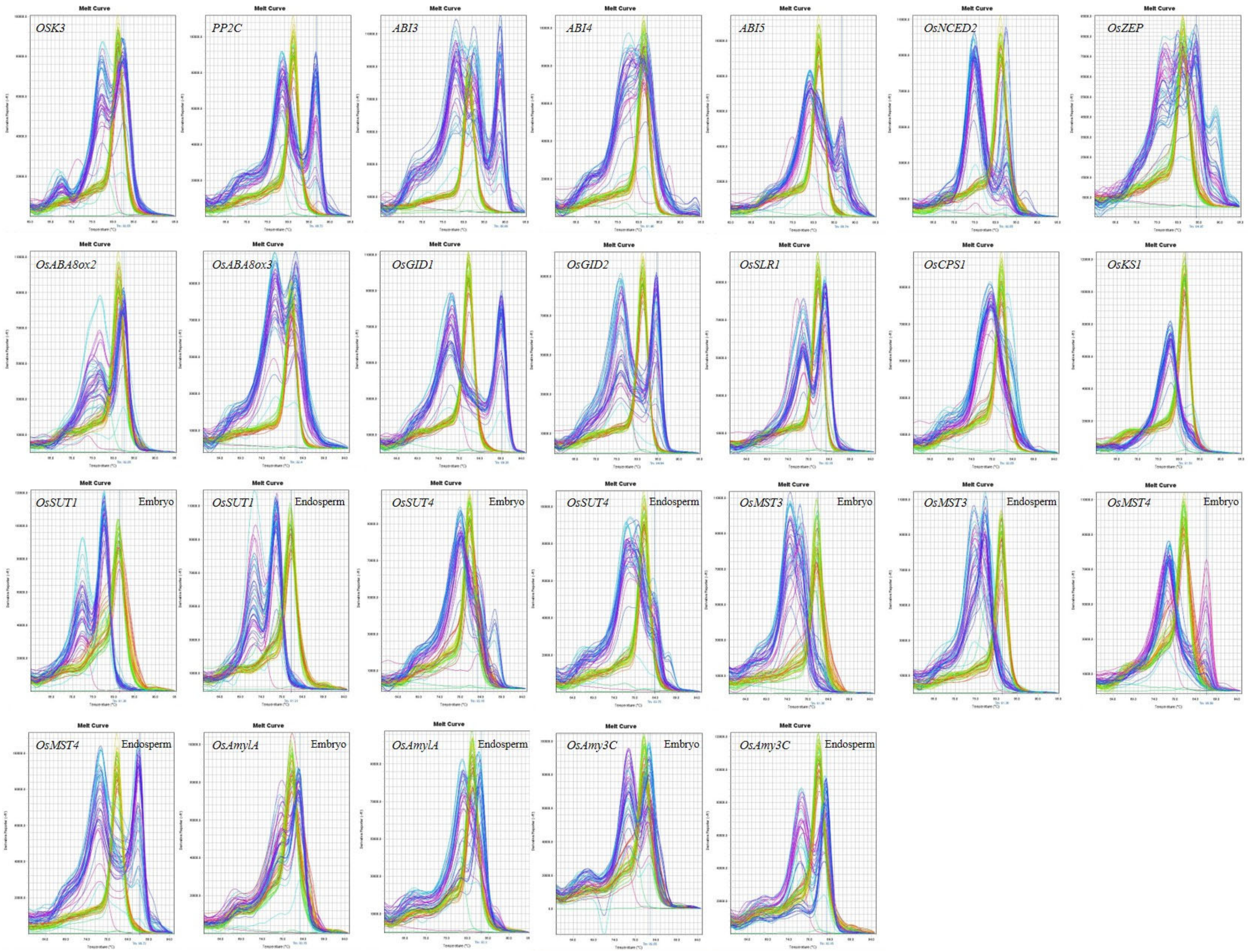

Figure S3. Representative melt curve profile of qRT-PCR products for all analyzed genes. For genes expressed in both the embryo and endosperm (*OsSUT1*, *OsSUT4*, *OsMST3*, *OsMST4*, *OsAmyA*, and *OsAmy3C*), melt curves from both tissues are presented; therefore, duplicate profiles appear in the figure.
